# Successful Use of Stellate Ganglion Block and Pulsed Radiofrequency in the Treatment of Posttraumatic Stress Disorder: A Case Report

**DOI:** 10.1155/2010/963948

**Published:** 2010-05-24

**Authors:** Eugene Lipov

**Affiliations:** Advanced Pain Centers S.C., 2260 West Higgins Road, Suite 101, Hoffman Estates, IL 60169, USA

## Abstract

*Objective*. To report our successful treatment of acute symptoms of posttraumatic stress disorder (PTSD). By the use of stellate ganglion block (SGB) and pulsed radiofrequency (PRF) to the stellate ganglion(SG) , sequentially. *Background*. A 48-year-old male a victim of armed robbery , who presented with extreme symptoms consistent with the diagnosis of PTSD. He was treated with antianxiety medications, as well as psychotherapy, but his symptoms persisted. *Methods*. Fifty-five days post trauma, we administered a SGB to the patient. One month later, we administered PRF to the right SG . We repeated the pulsed radiofrequency 30 weeks post trauma and performed a second SGB two weeks after that. *Results*. After the SGB , the patient experienced a major reduction in anxiety. Over the next week his improved allowing a significant reduction of antianxiety medications. One month later the symptoms returned and again subsided substantially following PRF , and that relief lasted four months. The patient than required another following PRF and a SGB with good responses. *Conclusion*. We report that selective blockade of the stellate ganglion via injection and the treatment with PRF, relieved our patient's symptoms of PTSD. And we also provide a plausible explanation of the effect.

## 1. Introduction

Posttraumatic stress disorder (PTSD) is a pathological anxiety that occurs when an individual experiences or witnesses severe trauma that constitutes a threat to his/her physical integrity or that of another person. The individual initially responds with intense fear, a sense of helplessness, or horror; later he or she may reexperience the event, with resultant symptoms of numbness, avoidance, hypervigilance, and hyperarousal. These symptoms lead to clinically significant distress and/or functional impairment [1]. 

The reported incidence of PTSD is increasing—in part, due to improved recognition, but also as a result of recent large-scale military and civilian traumatic events in the world. Despite the fact that PTSD is the most commonly diagnosed service-related mental disorder among U.S. military personnel returning from Iraq and Afghanistan, an expert panel convened by the Institute of Medicine found little evidence for the efficacy of most currently employed PTSD treatment modalities [2]. Because of its prevalence and the severe disability that can be associated with it, there is an urgent need for effective PTSD treatments. 

## 2. Case Report

A 48-year-old male was the victim of an armed robbery at the auto parts store he manages. The patient reported that robbers assaulted him as he was closing the store for the night, tied him up in the back of the store, held a handgun to his head, and hit him “with the gun over 100 times in the head.” He sustained minor, closed soft-tissue injuries, swelling, and bruising, which healed without treatment. One week post trauma the patient presented to his primary care physician with severe anxiety. He denied physical pain but reported having sporadic attacks of nausea, shaking, loss of appetite, and insomnia. He was prescribed escitalopram 10 mg/day, alprazolam 0.5 mg 4–6 times/day, and olanzapine 20 mg at bedtime.

Twenty-three days post trauma, the patient complained that the medications had not provided much relief. At that time, he underwent evaluation by a neuropsychologist with expertise in PTSD. During the assessment, the patient described feeling as if he had “too much adrenalin”, reported having intermittent chest tightness (“like I'm having a heart attack”), said he was still experiencing insomnia and nightmares, and mentioned that he had resumed smoking cigarettes (10 to 15 per day), after having quit smoking ten years earlier. Based on these complaints and a thorough medical/psychiatric history, the neuropsychologist diagnosed PTSD. He noted that the patient's presentation was tearful and marked by extreme anxiety and vigilance, which included motoric agitation, poor eye contact, restlessness, and distractibility. He recommended psychotherapy, which the patient agreed to. 

Owing to the severity of his physiological symptoms of anxiety, the patient's psychotherapy (with the neuropsychologist who evaluated him) began with relaxation training, which included listening to a progressive relaxation tape at home a few times a day. The patient found the tape helpful at first, but after a few weeks he experienced no further gains and said that he continued to have days when he felt like he was “going 110 miles per hour.”

The patient continued in psychotherapy, but his anxiety worsened. He was referred to our pain center as a potential alternative to psychiatric admission. Fifty-five days post trauma, with the patient's full written consent, we administered a right-sided stellate ganglion block. His neck was anesthetized with 2 cc lidocaine 2%. We then placed a 22-guage Quincke needle to the anterolateral aspect of the C6 vertebra under fluoroscopy. The needle contacted the bone and was pulled back 1 mm. This was followed by injection of 3 cc of iohexol (180 mg/mL) to visualize the ganglion and confirm needle placement, as well as to rule out intravascular, subarachnoid, or other inappropriate spread. Then 7 cc of 0.5% bupivacaine was injected next to the stellate ganglion to produce a sympathetic block. We monitored the patient's hand temperature during the anesthetic administration and noted a 2°C increase in the right hand; he also had facial anhidrosis and Horner's syndrome (i.e., enophthalmos, ptosis, swelling of the lower eyelid, miosis, and heterochromia)—all are evidence of a successful block. 

Immediately following the procedure, the patient said that he felt no anxiety. Over the next month, he kept a diary of his symptoms.

On Day 1 post SGB, the patient reported having some difficulty concentrating. He went out to dinner that evening with family and said that he “ate more food that night than I had eaten in a week.” That night he slept for 4 continuous hours. 

On Day 3, he had an episode of chest tightness, which was alleviated by alprazolam. He saw his psychologist, who was struck by a marked reduction in the patient's vigilance. He was “able to sit rather than having to stand and walk as he talked” and “had normal eye contact.” The patient commented, “At least I do not feel like I'm running at 110 miles per hour.” That night he slept soundly for 5 hours. 

He experienced two acute anxiety episodes per day on Days 4 and 5, which were relieved by alprazolam; he continued to eat well. On Day 6, he took 0.50 mg alprazolam upon awakening but did not take a second dose because he had an anxiety-free day.

One week post SGB, he reported 80% to 90% improvement in his anxiety level, 50% improvement in his appetite, and 25% improvement in his sleep. 

We saw the patient again on Day 32 (twelve and half weeks post trauma), at which time he complained that his symptoms had returned. In particular, he noted that his appetite was gone and his nightmares had come back. At that time, we obtained his written consent to administer pulsed radiofrequency (PRF) current to the right stellate ganglion. He was placed in a supine position on the procedure table with a shoulder roll. The right side of his neck was prepared with povidone-iodine and sterile drapes, and the skin was anesthetized. We then directed a 22-gauge, 10 cm insulated needle with a 10 mm, 42°C active tip to the sixth cervical vertebra, placing it in the anterior lateral position; this was confirmed by fluoroscopy. We used a 500 kHz current, applied at 2 bursts per second, with each burst lasting 20 milliseconds, for a total of 360 seconds. We monitored his right-hand temperature during the application, but no change was noted. After the procedure, he reported immediate relief (“a downshift from 5th to 1st gear”); his appetite improved that day, as did his sleep that night. One month later, the patient said that he was still “90% improved.” 

Four months following PRF, the patient's symptoms returned again. We repeated the PRF, and he experienced two weeks of relief. Then we repeated the SGB, which provided almost complete relief from most of his PTSD symptoms for one month; over the next few weeks he estimated that he was functioning at about 70% of his pretrauma level. Although the patient was on the verge of psychiatric hospitalization when he started his treatment with us, he has not been, nor has he needed to be admitted. 

## 3. Discussion

A literature search revealed no published reports on the use of either SGB or PRF in PTSD. The usual indication for SGB and PRF is complex regional pain syndrome (CRPS). Although CPRS was formerly known as reflex *sympathetic* dystrophy, recent studies point to *central *mechanisms for both its sensory and autonomic features [3]. PTSD is also to a large extent centrally mediated [1, 4, 5]. Grande et al. described the acute onset of CRPS in a patient with PTSD; they interpreted the CRPS as a stress response. The clinical association of the two conditions suggested shared neural substrate elements; they hypothesized that the patient's CRPS resulted from a dysfunctional relationship between the periaqueductal gray, a midbrain structure that coordinates defensive responses, and the anterior cingulate cortex in the forebrain, which is active in emotional processing [6]. 

### 3.1. Central-Sympathetic Connections

Our choice of interventions—sympathetic blocks targeting the stellate ganglion—was based partially on reports of successful endoscopic sympathetic block (ESB), that is, clipping of the sympathetic ganglia at the level of the second thoracic vertebra, for the treatment of severe anxiety and social phobia [7–9]. Telaranta, a pioneer of that treatment, has noted that social phobia and PTSD share many common features—most notably symptoms associated with overactivity of the sympathetic nervous system (SNS), such as heart racing, hypervigilance, and avoidance of painful psychic situations. [8]. At the same time, ESB of the thoracic ganglia and SGB are comparable sympathetic interventions. In fact, many of the efferent sympathetic fibers from the thoracic ganglia pass through the stellate ganglion [10], and the T2 ramus communicans is a known connection between the upper thoracic ganglion and the stellate [11]. Interestingly, both SGB and ESB have been used successfully for the treatment of schizophrenia, a condition at least partially characterized by exaggerated responses to external stimuli [1, 12, 13]. Based on these findings and insights, we speculated that manipulation of the neuronal circuitry in the neck connecting the SNS with key perception centers in the brain via SGB, then PRF, might reset those connections to a pretrauma state, thereby alleviating the patient's symptoms. 

Further objective evidence in support of these blocks comes from transsynaptic tracing methodology—the use of live viruses to label chains of neurons which has made it possible to delineate central efferents that regulate sympathetic targets [14, 15]. Utilizing pseudorabies virus injections in rats, Westerhaus and Loewy found that four cortical regions—insular, infralimbic, ventromedial temporal, and ventral hippocampal—were linked via multisynaptic connections to three sympathetic targets—the stellate ganglion, adrenal gland, and celiac ganglion [16]. The authors speculated that dysfunction in those central-sympathetic circuits may lead to exaggerated autonomic responses, including the extreme anxiety associated with PTSD [16]. 

The insular cortex has been shown to play a pivotal role in the pathophysiology of CRPS and PTSD [3, 5, 17]. Using voxel-based morphometry, Chen et al. demonstrated that survivors of a fire disaster with diagnosed PTSD had reduced gray matter volume in the insular cortex as well as in the hippocampus and anterior cingulate cortex compared with survivors of the same fire without PTSD [4]. Animal research related to PTSD has shown that repeated stress causes basolateral amygdala neurons to increase in dendritic complexity and sprout new synapses [5]. The hippocampus, which plays a role in memory and learning, receives input from the amygdala, and its function in spatial memory is altered by amygdala activity [5]. These neural changes may contribute to the altered autonomic responses (including increased fear) seen in PTSD. Positron emission tomography (PET) studies of patients with PTSD have shown dysfunction in the prefrontal cortex during symptom provocation and in response to traumatic reminders [18]. Bremner has suggested that dysfunction in the hippocampus and prefrontal cortex, both consistently implicated in PTSD, may underlie memory deficits and pathological emotions in PTSD [18]. 

### 3.2. SNS Dysfunction

PTSD can lead to overactivity of the adrenal glands [19], which is consistent with our patient's reports of “too much adrenalin” and “going 110 miles per hour.” As Westerhaus and Loewy showed, the adrenal glands and stellate ganglion have common connections with the cerebral cortex [16]. The hypervigilance associated with PTSD may be due to overactivation of the adrenals in particular and of the SNS in general. We speculate that SGB and PRF may reduce the adrenal output and relieve symptoms via a “rebooting” of the aforementioned central-sympathetic connections. 

Sleep disturbance is common in PTSD and was a recurring complaint of our patient. The cause may be a melatonin-related mechanism associated with the sympathetic system. Uchida et al. proposed that melatonin-related dysrhythmias, including sleep disorders, may be the result of chronically increased sympathetic nerve tone, which eventually causes decreased functioning of pinealocytes and reduced plasma melatonin levels. SGB, they suggest, may interrupt the sympathetic cycle, thereby allowing the normal melatonin rhythm to be reestablished [10].

### 3.3. PRF As an Alternative to SGB

PRF has gained popularity as an alternative to conventional radiofrequency (RF) treatment and is increasingly used to manage pain syndromes [20–22]. Unlike RF, which delivers continuous high-temperature (~85°C) current, PRF delivers high-intensity current in pulses; during each cycle, an active phase of 20 milliseconds is followed by a silent phase of 480 ms, which allows heat to dissipate, so that neurodestructive temperatures are not reached [22]. 

PRF may be used in place of local anesthetic in a variety of nerve blocks—including SGB—and usually provides longer-lasting effects [23]. Although its mechanism has not been fully elucidated, Cahana et al. concluded, after reviewing all the published clinical and laboratory data on PRF, that it involves “a genuine neurobiological phenomenon altering pain signaling” [21]. In the same publication Dr. Cahana reported that in his experience and literature review no complications have been associated with the use of pulsed radiofrequency. This was the reason for the substitution of PRF for SGB after one local anesthetic block in our patient. The usual convention is to do multiple Stellate Ganglion Blocks prior to a neurolytic or more permanent block. However we believe that the PRF is not neurolytic, is less dangerous than SGB, and may have longer effects.

 One theory for PRF's effects focuses on the upregulation of c-Fos, a marker of neuronal activity. In rats, PRF activates c-Fos in the dorsal horn; this expression is seen immediately and for up to seven days post PRF—far longer than would be expected as a direct effect of the applied current [21, 24]. The duration of c-Fos immunoreactivity may be the result of the inhibition of excitatory C-fiber responses [24]. Another theory is that PRF creates an electromagnetic field that depolarizes and disrupts nerve cell membranes via selective deactivation of small-diameter nerve fibers [21]. 

### 3.4. Right- versus Left-Sided Blocks

We administered a right-sided SGB and subsequently PRF to the right stellate ganglion because right-sided blocks affect right hemisphere structures, and, pertinent to PTSD, it is well established that the right hemisphere is responsible for producing autonomic responses to emotional stimuli, and the right amygdala is critically linked to unconscious emotional memories, whereas the left hemisphere is not involved in those processes [25]. PET studies of Vietnam veterans with combat-induced PTSD have demonstrated increased regional cerebral blood flow (rCBF) in the right amygdala [26]; moreover, PTSD symptom severity appears to be positively related to rCBF in the right amygdala [27] but not to blood flow in the left amygdala. Imaging studies have also shown that patients with PTSD have significant reductions in right hippocampal volume [18, 28, 29] and that neuronal density in right-sided medial temporal structures is significantly reduced [29]. 

## 4. Conclusion

Multiple CNS structures that are neuronally connected to the SNS appear to play a role in the onset and maintenance of PTSD. Recent advances in functional MRI and labeling techniques have enhanced our understanding of the neuroanatomy and allowed us to formulate a plausible explanation for the central effects of SGB and PRF in a patient with acute PTSD. It is important to note that, unlike existing treatments for PTSD, SGB and PRF provide virtually immediate results. We believe that further investigation of these interventions is warranted, as they may provide welcome alternatives and/or complements to current psychological and pharmacological treatments for PTSD, an increasingly prevalent condition in both military and civilian populations. We would like to introduce a new term “Chicago Block” which would designate that use of a cervical sympathetic block on the right side at C6 level. Technically stellate ganglion block is dune at C7 (one cervical level lower) and bilateral.
